# Modeling Realistic Clay Systems with *ClayCode*

**DOI:** 10.1021/acs.jctc.4c00987

**Published:** 2024-10-15

**Authors:** Hannah Pollak, Matteo T. Degiacomi, Valentina Erastova

**Affiliations:** † School of Chemistry, University of Edinburgh, Joseph Black Building, David Brewster Road, Edinburgh EH9 3FJ, United Kingdom; ‡ Department of Physics, Durham University, South Road, Durham DH1 3LE, United Kingdom; § UK Centre for Astrobiology, School of Physics and Astronomy, University of Edinburgh, James Clerk Maxwell Building, Peter Guthrie Tait Road, Edinburgh EH9 3FD, United Kingdom

## Abstract

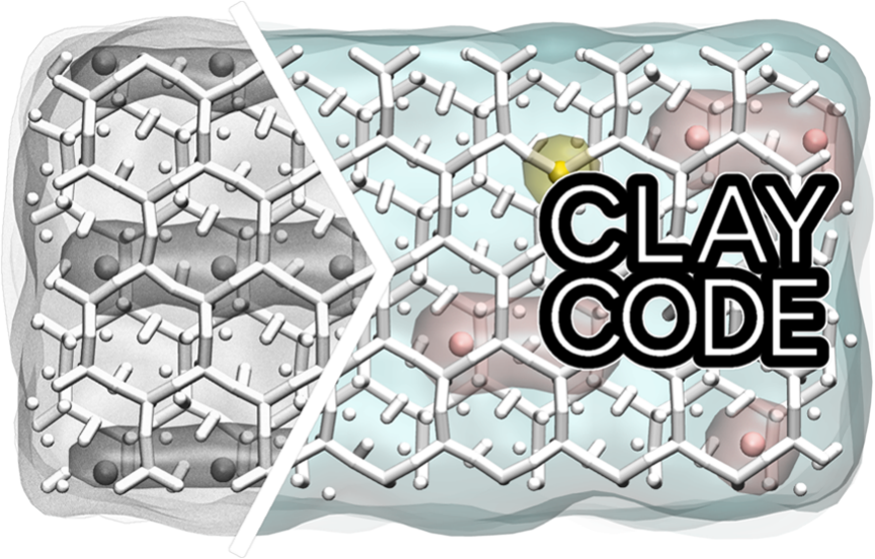

Clays are a broad class of ubiquitous layered materials. Their specific chemophysical properties are intimately connected to their molecular structure, featuring repeating patterns broken by substitutions. Molecular dynamics simulations can provide insight into the mechanisms leading to the emergent properties of these layered materials; however, up to now, idealized clay structures have been simulated to make the modeling process tractable. We present *ClayCode*, a software facilitating the modeling of clay systems closely resembling experimentally determined structures. By comparing a realistic model to a commonly used montmorillonite clay model, we demonstrate that idealized models feature noticeably different ionic adsorption patterns. We then present an application of *ClayCode* to the study the competitive barium and sodium adsorption on Wyoming montmorillonite, Georgia kaolinite, and Montana Illite, of interest in the context of nuclear waste disposal.

## 1 Introduction

Clay minerals are ubiquitous on Earth and are also found on other rocky planetary bodies. They are typically formed through prolonged chemical weathering of silicate-bearing rocks in the presence of water or during hydrothermal activity. Therefore, clays are a key component of soil and are crucial for various geological processes, including soil formation, weathering, and diagenesis. Furthermore, clay minerals play a central role in industrial and environmental applications, such as improved soil fertility, contaminant management, mining, and water purification. Clay mineral structures are affected by environmental conditions during their formation, which leads to a large variety of mineral structures with a broad range of properties. To predict the behavior of clay minerals in changing environments or during an industrial application, we must understand the relationship between their structure and their properties.

The development of molecular simulations over the last half a century has brought atomistic-level insights into the structural and dynamic properties of molecular systems. The methods applied to the study of clay minerals have been ranging from quantum mechanical simulations–studying electronic structures, properties and chemical processes; to molecular mechanics–where atoms are represented as classical spheres allowing for simulation of large atomic systems and dynamic processes; to mesoscale levels–where coarse-graining groups of atoms into beads further increase accessible simulated system’s scales.^1−4^ The choice of method and availability of computational resources impose limitations on the phenomena one can model. Quantum calculations are essential to study chemical processes at the mineral interface,^4,5^ to refine mineral structures and understand local chemical environments,^6^ and to predict their spectroscopic properties.^7^ These calculations typically involve a few hundred atoms and are on picosecond time scales.^5^ Therefore, these are less suited to study emergent geological properties and phenomena associated with longer size- and time scales. Quantum calculations are, however, key to the parametrization of classical force fields,^8−10^ and, more recently, machine-learned potentials.^11,12^ These classical models enable observing phenomena such as the diffusion of fluids and solutes at mineral surfaces,^13,14^ or collective motions such as clay swelling or aggregation.^15^ In this context, molecular dynamics (MD) is the sampling scheme most commonly used.

While the number of publications utilizing MD simulations for clay studies is growing (see Figure S1, SI), the variety of clay models studied is still very limited and nohow representative of the broad array of naturally occurring clay minerals. This deficit can be only partially attributed to the narrow range of force field parameters currently available. Indeed, one of the key difficulties lies in the preparation of the clay model itself for the simulation, which is a time-consuming and error-prone procedure.

To facilitate the model setup and simulation of clay minerals representative of their natural counterparts, we have developed *ClayCode*github.com/Erastova-group/ClayCode. This software enables the preparation of clay models closely matching their experimentally determined counterparts, and the assignment of ClayFF force field^9,16^ parameters. All input files necessary for simulating the resulting parametrized model with GROMACS,^17^ one of the fastest and most commonly used engines, are finally produced.

In this paper, after an overview of clay structures, we review the current state-of-the-art in the molecular modeling of clays, the tools available to assist users in the preparation of simulations, and their limitations. We then detail the *ClayCode* workflow, highlighting how its flexibility and modularity enable its applicability to a broad range of molecular systems. To demonstrate the importance of modeling realistic clay systems, we compare adsorption of metal ions on two simulated models of Wyoming montmorillonite—a common simple model previously used by us^18,19^ and other researchers^20,21^—and a new model produced by *ClayCode*, truthful to the experimentally characterized structure of the mineral. Our results demonstrate that the subtle structural differences observed in natural clay minerals are key determinants of the mineral’s physical properties. Finally, we demonstrate the usage of *ClayCode* in a real-case-scenario by comparing the adsorption of two metal ions, Na^+^ and Ba^2+^, on three natural clays: Wyoming montmorillonite (SWy-1), Georgia kaolinite (KGa-1), and Montana illite (IMt-1). The choice of these ions is driven by their properties, with sodium being among the most common monovalent cations in nature, and barium being a divalent heavy metal nuclear fission product, a component of nuclear waste used as a laboratory analogue to the more dangerous radioactive radium.^22−24^

### 1.1 Why Clay Mineral Structures Are So Interesting?

Typically, the term clay mineral refers to hydrous phyllosilicates, further classified based on the chemophysical characteristics (see Table 1) arising from their structures. Since this work focuses on developing representative atomistic models of clay minerals, we must first review their structures and associated nomenclature.

**Table 1:**
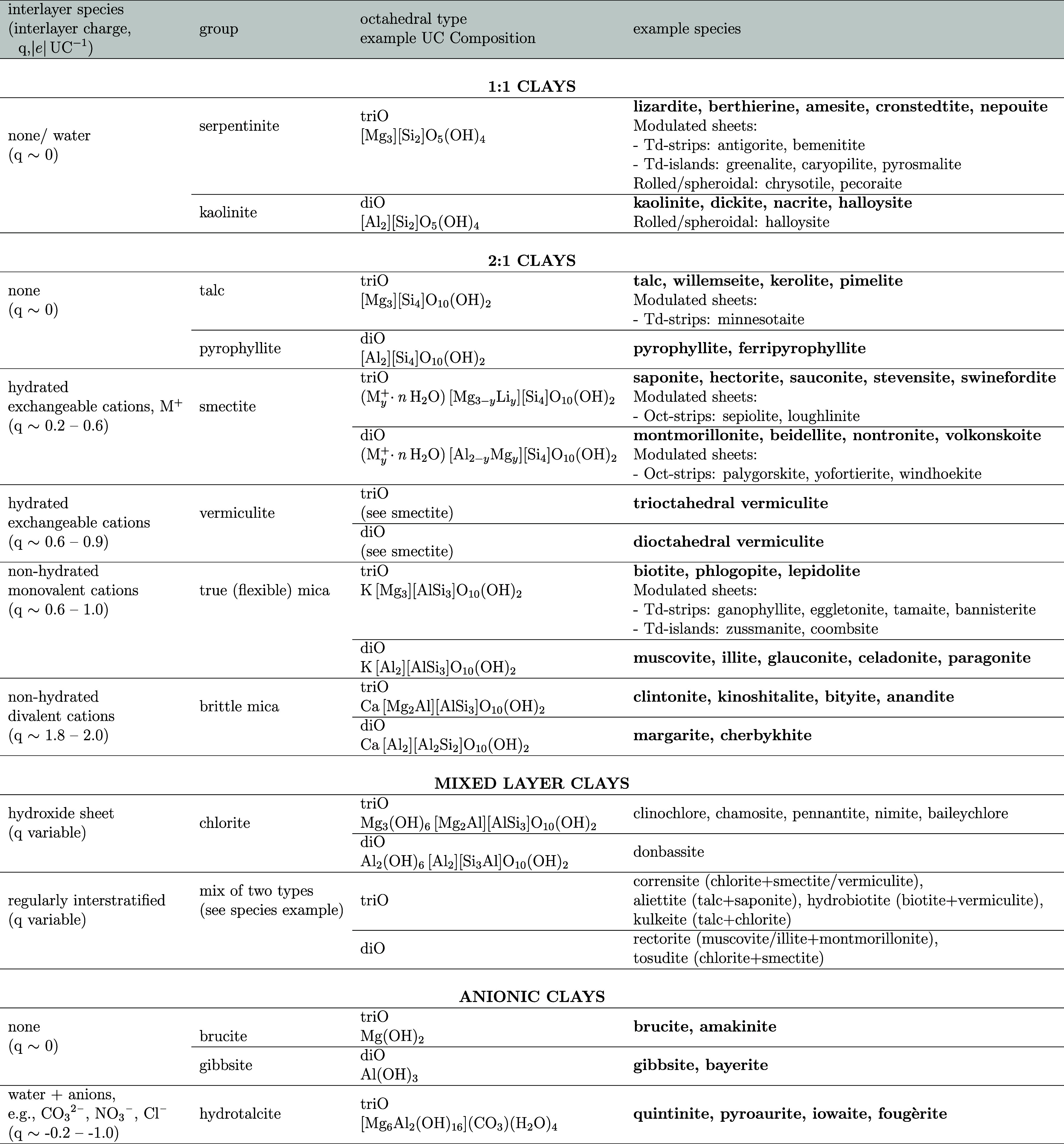
Classification of Clay Minerals, Including Phyllosilicates and Layered Double Hydroxides^a^

a Partially adapted from Martin et al.^25^ The example clay species are all planar unless otherwise specified, the example species that can be constructed with *ClayCode* are highlighted in **bold**. UC - unit cell; triO - trioctahedral; diO - dioctahedral.

**Figure 1:**
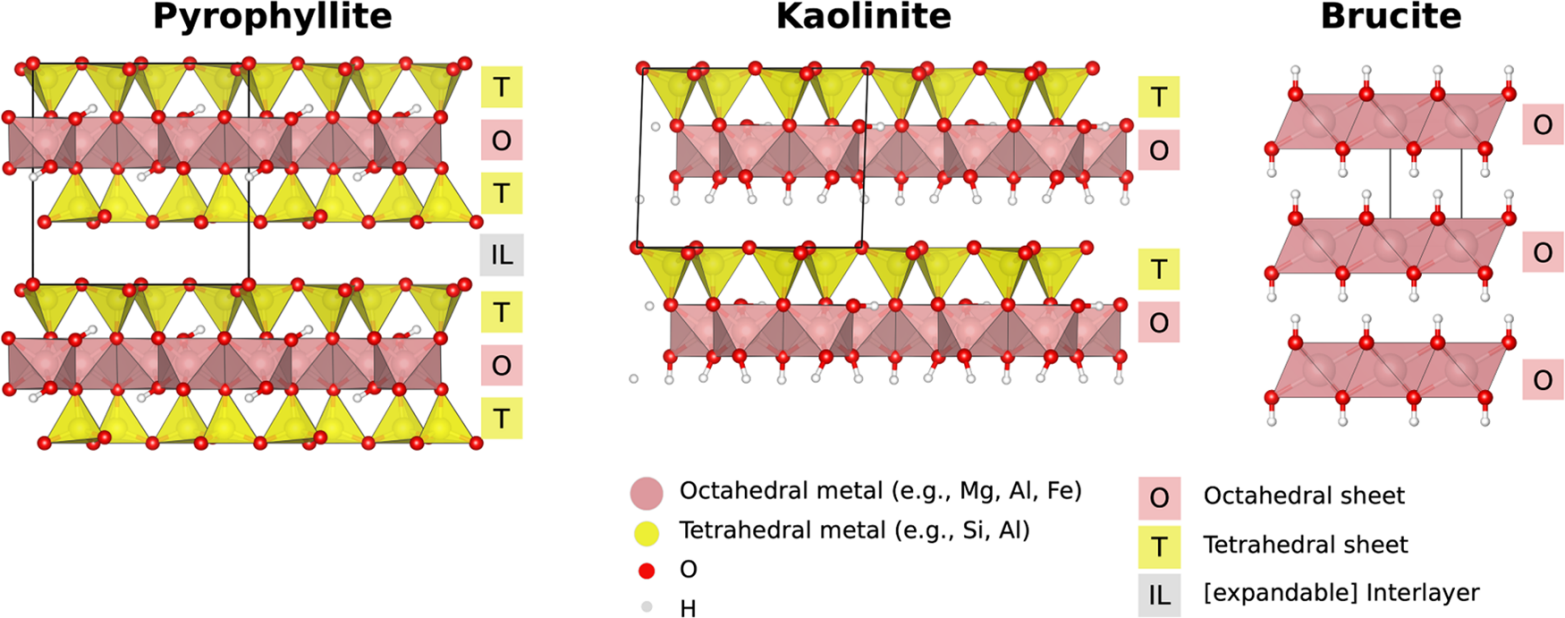
View onto three example representative clays: 2:1 pyrophyllite, 1:1 kaolinite and anionic clay brucite. The unit cell is shown in black, T sheet in yellow, O sheet in pink, and interlayer (IL) space marked in gray.

At an atomic level, clay minerals are layered sheet silicate minerals. Each layer is composed of a stack of tetrahedral (T) sheets bridging to octahedral (O) sheets. T sheets are made up of a hexagonal network of SiO_4_-tetrahedra, which are connected via three of their four oxygen atoms, the remaining apical oxygen links to the O sheet. O sheets feature octahedra of 6-fold divalent or trivalent metal cations (M^2+/3+^), e.g., Al^3+^, Fe^3+^, Fe^2+^ or Mg^2+^. The O sheet metal ion valency is M^3+^ in dioctahedral (diO) and M^2+^ in trioctahedral (triO) clays. In diO clays, only two out of three sites within the O sheet are occupied. Depending on the vacancy position relative to the hydroxyl groups, there exist *cis*- and *trans*-vacant varieties. T and O sheets are connected via the apical oxygen atoms of the T sheet bridging to the octahedral metal cations. If the O sheet is only bound to one T sheet, the clay has a 1:1 structure (TO), whereas if the O sheet is sandwiched between two T sheets, the clay is of 2:1 type (TOT). Clay mineral geometries are usually defined via unit cells (UCs), where one UC represents the smallest periodically extendable unit of a clay layer. Figure 1 presents schematics of possible UCs, and Table 1 details example UC compositions.

In a neutral clay, all T sites are occupied with Si^4+^, and O sites contain M^2+^ metal cations for triO, or M^3+^ for diO clays (e.g., pyrophillyte). Permanent negative layer charges arise from isomorphic substitutions (T: Si^4+^ → M^3+^, diO: M^3+^ → M^2+^, triO: M^2+^ → M^1+^) or vacancies. This charge is compensated by cations in the interlayer space.

Systems with low to medium layer charge (−0.2 to −0.6 |*e*| UC^–1^) are swelling clays (e.g., smectite). Their interlayer is populated by hydrated cations which are exchangeable for other ions or molecules. Swelling occurs through an exchange of interlayer species or varying hydration of the interlayer, leading to an expansion or contraction of the *d*-space (defined as the sum of the clay layer thickness and interlayer space). Initial expansion proceeds in discrete steps that correspond to the formation of up to four water layers (*d*-space from 10 to 22 Å), and is referred to as crystalline swelling. Beyond this distance (*d*-space >22 Å), adjacent clay layers are no longer coordinated. Any further continuous increase in separation is called osmotic swelling.

At high layer charges, between −0.6 to −0.9 |*e*| UC^–1^, the electrostatic attraction between clay and interlayer cations is too strong to permit an expansion beyond one to two water layers (e.g., vermiculite and true mica). As layer charges increase even further, reaching up to −1.0 |*e*| UC^–1^ for brittle mica, clay-cation attractive forces become too strong for any swelling to occur.

Unlike 2:1 clays formed by TOT layers, i.e., always exposing T sheets to the interlayer, the 1:1 clays are made of TO sheets and expose two different types of surfaces: a hydrophobic siloxane and a hydrophilic hydroxide (see kaolinite on Figure 1). Typically, 1:1 clays do not feature large amounts of isomorphic substitutions and, therefore, do not have a permanent charge.

Another class of minerals we must mention is layered double hydroxides (LDHs) (see brucite on Figure 1). Although technically not clay minerals, LDHs share many of their properties including a layered structure, isomorphic substitutions producing variable layer charge density, ion-exchange properties, and the ability to swell and intercalate. LDHs only feature octahedral sheets, where substitutions produce a positive charge. For example, in a hydrotalcite Mg^2+^ may be replaced by Al^3+^, giving rise to a positive charge that is often counterbalanced by a CO_3_^2–^ anion in the interlayer. For this reason, LDHs are sometimes referred to as anionic clays. While present in nature, LDHs are also commonly synthesized in a laboratory setting. Since control over their composition is straightforward, it is commonplace to name them by only stating the ratio of M^2+^ to M^3+^. For instance, an hydrotalcite with a [Mg_6_Al_2_(OH)_16_](CO_3_)(H_2_O)_4_ composition can be simply named 3:1 MgAl-LDH.

Table 1 summarizes clay classification, listing the core nine groups of silicate clay minerals as well as anionic clays, alongside example mineral species. It can be seen that not all clays are planar: some involve modulated sheets connecting via either O or T sheets, and some are rolled. While fascinating, these cases are relatively uncommon and thus are not the focus of the current discussion, and are not currently implemented in *ClayCode*. Most importantly, with this summary, we wish to emphasize the vast variety of clay species originating from the small changes in the clay structure.

For instance, montmorillonite is among the most widely studied clays, thanks to its powerful adsorption abilities and common presence in soils around the world. Well-characterized samples can be purchased from the Clay Minerals Society^26^ for experimental work. Within these samples there exist a few subtypes:

1. Texas montmorillonite (STx-1):
  [[Si_8.00_][Al_2.41_Fe(III)_0.09_Mn_<0.01_Mg_0.71_Ti_0.03_]O_20_(OH)_4_]^−0.68^
2. Wyoming montmorillonite (SWy-1, SWy-2, SWy-3):
  [[Si_7.98_Al_0.02_][Al_3.01_Fe(III)_0.41_Mn_0.01_Mg_0.54_Ti_0.02_]O_20_(OH)_4_]^−0.55^
3. Otay montmorillonite (SCa-2):
  [[Si_7.81_Al_0.19_][Al_2.55_Fe(III)_0.12_Mn_<0.01_Mg_1.31_Ti_0.02_]O_20_(OH)_4_]^−1.48^

Yet, this diversity has not been reflected in the simulations up to now. Indeed, montmorillonite is commonly simulated using one of the following four idealized structure models:

1. [[Si_8_][Al_3_Mg_1_]O_20_(OH)_4_]^−1.00^ ^18−21^
2. [[Si_7.75_Al_0.25_][Al_3.25_Mg_0.75_]O_20_(OH)_4_]^−1.00^ ^27^
3. [[Si_7.75_Al_0.25_][Al_3.5_Mg_0.5_]O_20_(OH)_4_]^−0.75^ ^28−33^
4. [[Si_8_][Al_3.25_Mg_0.75_]O_20_(OH)_4_]^−0.75^ ^21,34−37^

### 1.2 What Tools Are Available and Why Do We Need Another?

Determining the structure and parameters of a clay model is a complex and laborious process. This task has to be accomplished either manually or with software tools of limited applicability, which explains why to date clay models used in simulations are greatly simplified. Historically, the most common tool used to prepare MD simulations of clay systems has been the commercial software Materials Studio.^38^ This software enables the user to create custom material structures via a graphical user interface and offers tools to help assign force field parameters. While useful, Materials Studio’s applicability is limited by the ability of the user to accurately draw out the desired clay or load in the desired CIF files, reassigning the atoms by hand. Furthermore, its force field assignment needs to be manually checked and validated, which is itself a laborious process.

In the last five years, two freely available tools have also become available. The *atom*(39) library leverages on MATLAB to enable setting up a wide range of periodic inorganic structures. While flexible, it requires programming knowledge, and cannot be used as a standalone application. The more recent CHARMM-GUI *Nanomaterial Modeler*,^40^ while more accessible than *atom*, can only handle four simplified clay species (montmorillonite, kaolinite, pyrophyllite, and muscovite).

The *ClayCode* Python package automates the setup of clay models for classical MD simulations, offering tools to design realistic clay models and to set up custom simulation workflows. *ClayCode* has been designed to be user-friendly: while its behavior is fully customizable, its default parameters enable simulation of most common systems with minimal user intervention. In its current embodiment, *ClayCode* enables modeling planar hydrated clay systems ready for simulation with GROMACS.^17^ Clay sheets are assembled from UCs modeled with the mainly nonbonded ClayFF force field parameters.^9,16^ By default, water and ions are described according to the simple point charge (SPC) model^41^ and ion parameters by Smith et al.;^42^ however, the user can choose alternative models. Table 2 provides a side-by-side comparison of CHARMM-GUI *Nanomaterial Modeler*, *atom*, and our *ClayCode*.

**Table 2:** Comparison of CHARMM-GUI *Nanomaterial Modeler*, *atom,* and *ClayCode*^a^,^b^

	*Nanomaterial Modeler*	*atom*	*ClayCode*
language	Tcl/Tk	MATLAB	Python
			
force field	INTERFACE	ClayFF	ClayFF
		INTERFACE	
			
simulation engine compatibility	AMBER	GROMACS	GROMACS
	CHARMM	NAMD*	
	GENESIS	LAMMPS*	
	LAMMPS	RASPA2*	
	NAMD		
	OpenMM		
			
UC database provided	[Al_4_][Si_4_]O_10_(OH)_8_	no	list in Table 3
	diO 1:1 kaolinite		

	[[Al_4–*m*_Mg_*m*_][Si_8–*n*_Al_*n*_]O_20_(OH)_4_]^−(*m*+*n*)^		
	*cis*-vacant diO 2:1		
	pyrophyllite (*n*=0, *m*=0),		
	montmorillonite (*m*≠0, *n*=0),		
	muscovite (*m*≠0, *n*≠0)		
			
user UC input	not possible	yes	yes

a * indicates that compatibility is available for ClayFF only.

b UC – unit cell.

## 2 The *ClayCode* Workflow

*ClayCode* runs in the terminal of Unix-based operating systems. It automatically builds and prepares for the simulations periodic atomistic models of hydrated planar clay sheets using an internal customizable database of UCs. To this end, the *ClayCode* workflow is subdivided into a set of steps, handled by independent modules, explicitly designed to facilitate the addition of new functionalities. Currently, the available modules are data (with an internal UC and force field database where custom UCs and force fields can be added), builder (to assemble the clay system), and siminp (to generate associated GROMACS input files). The user can then analyze the produced GROMACS trajectories with typical MD analysis tools, e.g., GROMACS own ones,^17^ MDAnalysis,^43^ VMD,^44^ or our own DynDen.^45^ The user interacts with each *ClayCode* module via plain text files in YAML format. Hereafter, we describe the main functionalities of each module. A full list of all available keywords, along with their default values, can be found in the online documentation at claycode.readthedocs.io.

### 2.1 Handling Unit Cells with the data Module

Clay sheets are made from an internal UC database containing a selection of different unit cell types that have been either constructed from the American Mineralogist Crystal Structure Database (AMCSD) crystallographic data or taken from our previous works (see summary in Table 3). The availability of preassigned UCs allows constructing a large number of clays without the need to dwell into crystallographic data and assigning ClayFF atom types to the UCs. Nevertheless, the user can also expand the database as needed.

**Table 3:** Currently Available Unit Cell Types in *ClayCode* UC Database^a^

UC type	UC description	AMCSD code	example	refs
TD21	*trans*-diO 2:1	0007180	nontronite	(50)
CD21	*cis*-diO 2:1	0002868	montmorillonite	(18,51)
TD11	*trans*-diO 1:1		dickite	
CD11	*cis*-diO 1:1		kaolinite	(13,19,49)
T21	triO 2:1	0015819	saponite	(52)
T11	triO 2:1		lizardite	
LDH21	2:1 LDH		quintinite	
LDH31	3:1 LDH	0007912	hydrotalcite	(47,48,53)

a UC constructed from structures in the AMCSD,^46^ or manually curated by us for this work or in the earlier studies.^13,18,19,47−49^

Adding new UCs, based upon already assigned unsubstituted ones, can be done via the data module. In this step, UCs containing all possible substitutions are generated. This operation can be repeated iteratively, to produce UCs featuring up to three substitutions. For all generated UCs, as default the maximum accepted charge limit arising from isomorphic substitutions is set to −2.0 |*e*| UC^–1^. Any UC with substitution combinations resulting in higher charges will not be added to the database.

### 2.2 Setting up Clay Models with the builder Module

**Figure 2:**
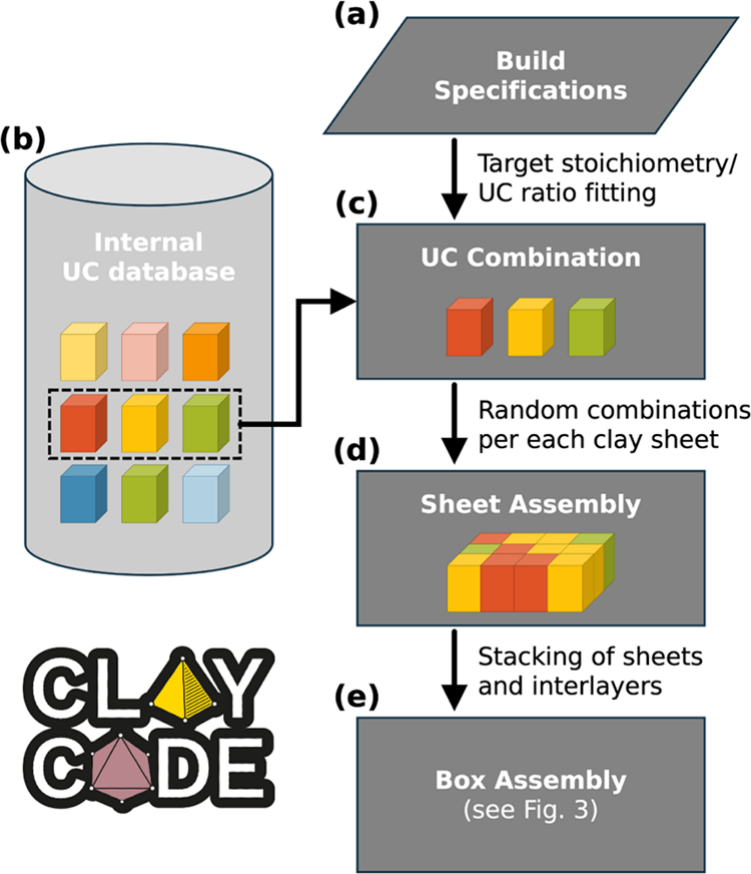
Illustration of sheet assembly workflow. Every clay system is made of individual clay sheets, each featuring a random arrangement of known UC in proportions to match user-defined specifications. *ClayCode* logo reproduced with permission.

A hydrated clay system is assembled according to a four-step pipeline, illustrated in Figure 2, ultimately yielding a topology and a coordinates file ready for simulation with GROMACS. Hereafter, we describe each step.

#### 2.2.1 Parsing and Checking User-Provided Input Data

The minimal input for model building requires a system name, the UC type, and either a specification of the average UC stoichiometry, or ratios of UC indices from which the sheets should be assembled (Figure 2a).

#### 2.2.2 Matching Target UC Composition

Using the UC database (Figure 2b), *ClayCode* will either find the combination of UCs that provides the closest match to a user-defined target stoichiometry, or compute the numbers of each UC that correspond to given UC ratios (Figure 2c). *ClayCode* only adds substitutions of elements with a minimum occupancy of 0.05 atoms per UC. In the case of substitution not available in the UC database, those under the threshold are removed and the remaining sheet occupancies are adjusted to match the average target charges. For the ones above the threshold, the user is prompted to specify the oxidation state of the incompatible atom type which is then also removed from the target UC charge.

#### 2.2.3 System Assembly

**Figure 3:**
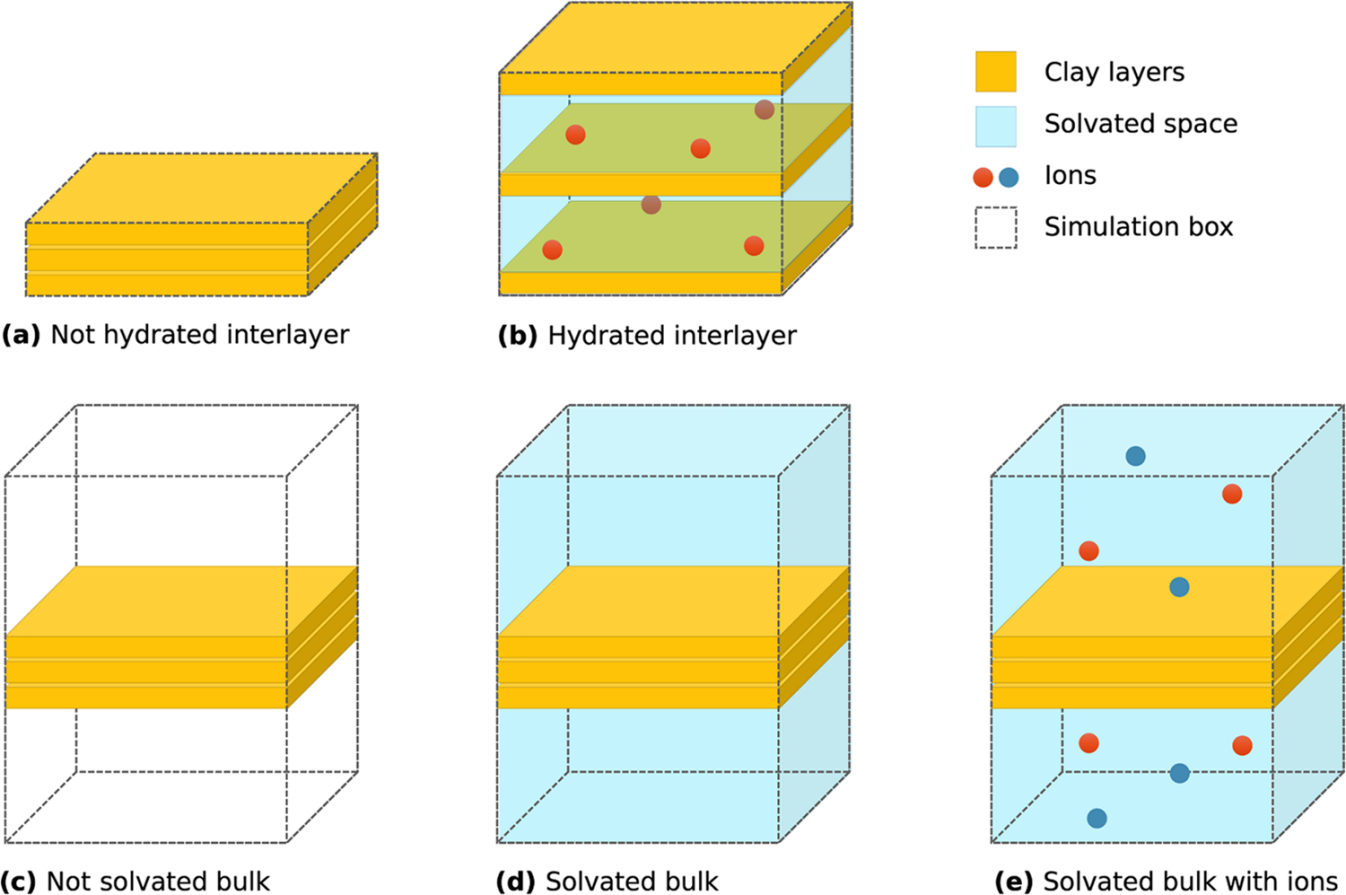
Illustration of clay models with different sheet stackings. Stacked clay sheets can either be without interlayer water (a) or with a hydrated interlayer (b). The different bulk space setup options also offer setup of a nonsolvated (c), solvated with water only (d) or with added ions (e) systems.

Sheets are assembled from a randomized sequence of the selected UCs (Figure 2d). This randomization is important to represent a realistic clay structure, where substitutions are randomly distributed within the sheets. Combinations of UCs yield clay sheets with substitutions that obey the Loewenstein rule.^54^ Then, the interlayer is generated using GROMACS solvate and genion tools. The sheets and interlayers are finally stacked, the layer charges resulting from isomorphic substitutions are compensated by counterions, as selected by the user; and, if specified, solvent and bulk ions are added to an extended simulation box bulk space (Figure 2e). Currently supported box arrangements are illustrated in Figure 3.

It has to be noted that the accuracy of a clay model is limited by the number of UCs within each sheet. In particular, to achieve a close match to a target composition with minor quantities of substitutions, a minimum sheet size is required. By default, *ClayCode* accepts absolute deviation of occupancies from the target structure below 0.025 atoms per UC.

#### 2.2.4 Energy Minimization

The final step is a GROMACS energy minimization run. All resulting output (topology, coordinates, and a log file reporting on *ClayCode* operations) is then placed inside an output folder with the system’s name.

### 2.3 Writing Simulation Run Scripts with siminp Module

The siminp module produces all the files required for custom molecular dynamics pipelines. To this end, the user specifies a series of run types and the GROMACS version that will be used to run the simulations. By default, *ClayCode* will produce an output to be run with the GROMACS version which is installed locally. This input is then processed by *ClayCode* and a directory tree with all required input files (coordinates, topology, force field and run parameters) and a run script is constructed. The run parameter MDP files are assembled based on the allowed parameters for the selected GROMACS version. Then, if specified, GROMACS default parameters are overwritten with run-specific user-defined MDP options.

One of the features of siminpis its ability to generate *d*-space equilibration run input files, whereby water molecules are iteratively removed from a hydrated interlayer until the distance between clay sheets converges to a specified distance.

## 3 Methods

### 3.1 Clay Models

KGa-1, SWy-1 and IMt-1 clay models were constructed using *ClayCode* to match experimental compositions of Clay Minerals Society source clays Georgia kaolinite KGa-1, Wyoming montmorillonite SWy-1/SWy-2/SWy-3 and Montana illite IMt-1/IMt-2, respectively. Clay sheets were assembled from UCs available within the database: CD21 UCs for SWy-1 and IMt-1, and CD11 UCs for KGa-1. Furthermore, we also produced a simplified montmorillonite model (hereon SWy-simp.) matching that used in our previous work.^18,19^ To this end, clay sheets were assembled from a single *cis*-diO D21 UC with one O substitution of Al^3+^ for Mg^2+^ per UC. This was done by selecting *ClayCode*’s UC ratio method rather than giving a target composition.

Table 4 summarizes the experimental and modeled clays’ stochiometries. KGa-1 and IMt-1 have nonexchangeable interlayer ions, therefore, those are matched to the reference structures. Montmorillonite is a swelling clay with exchangeable interlayer ions. Typically, in the laboratory, before starting adsorption studies, swelling clays undergo homoionisation. To replicate this, we have set the interlayer ions to be Ca^2+^. This interlayer space in swelling clays is variable and is influenced by the ionic composition and hydration levels. For a Ca-rich hydrated montmorillonite the *d*-space is generally around 1.5 nm, which is what we have also set here. The YAML files and the experimental composition file used to set up these systems are all included as Supporting Information. We note that, as the UCs arrangement is randomly generated, each new run of *ClayCode* will produce a slightly different model.

**Table 4:** Average UC Stoichiometries of Simplified (SWy-simp.) and Realistic SWy-1, KGa-1 and IMt-1 Models and Their Corresponding Experimental Reference (ref) Data from Clay Mineral Society^26^^,^^a^

clay system	average UC stoichiometry
SWy-simp. model	(Ca_0.48_Na_0.04_)[[Si_8_][Al_3_ Mg]O_20_(OH)_4_]^−1.00^
SWy-1 model	(Ca_0.25_Na_0.04_)[[Si_7.97_Al_0.03_][Al_3.02_Fe_0.43_^III^Mg_0.54_]O_20_(OH)_4_]^−0.54^
SWy-1 ref	(Ca_0.12_Na_0.32_K_0.05_)[[Si_7.98_Al_0.02_][Al_3.01_Fe_0.41_^III^Mn_0.01_Mg_0.54_Ti_0.02_]O_20_(OH)_4_]^−0.53^
KGa-1 model	(Mg_0.02_Ca_0.02_Na_0.05_K_0.04_)[[Si_3.83_Al_0.17_][Al_3.97_Fe_0.03_^III^]O_10_(OH)_8_]^−0.17^
KGa-1 ref	(Mg_0.02_Ca_0.01_Na_0.01_K_0.01_)[[Si_3.83_Al_0.17_][Al_3.86_Fe_0.02_^III^Mn_tr_Ti_0.11_]O_10_(OH)_8_]^−0.06^
IMt-1 model	(Ca_0.06_Mg_0.09_K_1.37_)[[Si_6.77_Al_1.23_][Al_2.74_Fe_0.03_^II^Fe_0.83_^III^Mg_0.40_]O_20_(OH)_4_]^−1.67^
IMt-1 ref	(Ca_0.06_Mg_0.09_K_1.37_)[[Si_6.77_Al_1.23_][Al_2.69_Fe_0.06_^II^Fe_0.76_^III^Mn_tr_Mg_0.43_Ti_0.06_]O_20_(OH)_4_]^−1.68^

a The interlayer ions are given in parentheses.

The produced clay models were then solvated with SPC water,^41^ the excess charge was neutralized with Na^+^ and Ba^2+^ ions.^42^ Then, the bulk ion concentrations were adjusted to equivalents of 0.1 mol L^–1^ of Na^+^ and Ba^2+^ by adding NaCl and BaCl_2_ in equimolar quantities. Practically, if the cation concentrations after charge balancing are above 0.1 mol L^–1^, no further ions were added. Average final simulation box dimensions, numbers of interlayer and bulk water, and numbers of inserted bulk ions are listed in Table S1, SI.

### 3.2 Molecular Dynamics Simulations

All of the simulations (energy minimization, equilibration and production runs) were carried out with GROMACS 2022.3.^17^ Energy minimization was performed at the end of the setup procedure with *ClayCode*, using the default parameters set by *ClayCode* – steepest descent algorithm using as convergence criterion the maximum force on any one atom being less than 500 kJ mol^–1^ nm^–1^.

This was followed by two short equilibration runs, each of 0.5 ns with a step size of 0.5 fs. The first one was performed in a Canonical (NVT) ensemble, with the Velocity-rescale thermostat set to 300 K and the clay layers fixed along *z*-direction. The second equilibration run was performed in the isothermal–isobaric (NPT) ensemble, adding Parrinello-Rahman barostat set to 1.0 bar with semi-isotropic scaling to allow the decoupling of *xy*-plane and *z*-axis of the simulation box.

For the swelling clay models, i.e., SWy-simp. and SWy-1, this was followed by a sequence of *d*-space equilibration runs, with the files generated with siminp module of *ClayCode*. To this end, a number of interlayer water molecules (here, 50 molecules) is removed, and then the system undergoes a short NPT equilibration that allows the *z*-axis to contract, accounting for the new hydration level. The *d*-space is then compared with a target value (here, 1.5 nm), and the dehydration step is repeated until an agreement is reached. For the systems in this work, it took six steps to obtain the desired *d*-space. Each equilibration run is 0.1 ns long with 1 fs time step, 300 K and 1.0 bar controlled with Nose-Hoover and semi-isotropic Parrinello–Rahman algorithms, respectively.

From there, the systems continue into production runs of 70 ns and a time step of 1.0 fs in the isothermal–isobaric (NPT) ensemble with and Nose-Hoover thermostat at 300 K and semi-isotropic Parrinello–Rahman barostat at 1.0 bar.

In all simulation runs, neighbor searching was performed every 10 steps, and electrostatic and van der Waals interactions were computed using particle mesh Ewald algorithm with geometric combination rules, Verlet cutoff-scheme and 1.4 nm cutoff distances. The LINCS algorithm was used for H-bond constraints.

Production run trajectories were written at 2 ps intervals. The last 50 ns of each simulation were used for analyses, after the systems were assessed for convergence with DynDen.^45^

### 3.3 Analysis

Linear number densities were calculated for each simulation using the GROMACS density tool, with a window of 0.01 Å. Densities were calculated independently for the clay (represented by all of its atoms), Ba^2+^, and Na^+^ atoms (see Figure S2, SI). We note that there is some asymmetry observed in these plots. The asymmetry is an effect of the two clay layers not being exact mirror images of one other and ions positions being randomly initialized, where proximity to a strongly interacting site will result in the ion being trapped. This was previously observed and discussed when simulating a realistic clay system.^55^ To accommodate for this phenomenon, observations for both sides of each clay can be aggregated.

All linear densities were analyzed with an in-house Python code. For each simulation, we identified the position of clay boundaries along the *z*-axis (perpendicular to the clay surface) as the maxima in the first and last peak in its linear density (hereon *z*_upper_ and *z*_lower_, respectively). We used these boundaries to curate the measured ionic number densities. Specifically, we removed all ion density located between *z*_lower_ and *z*_upper_, i.e., ions in the interlayer, so only the distribution of ions in solution were considered. Each normalized density was then split in two parts, describing ionic distributions above and below the clay. Ionic densities above the clay were defined as those associated with a *z*-position greater than *z*_upper_, and those below the clay as those with *z*-position lower than *z*_lower_. The resulting two density distributions were shifted to bring their *z*_upper_ and *z*_lower_ position to the origin, respectively. Finally, the two distributions were overlaid by mirroring that of upper ions. SWy-simp., SWy-1, and IMt-1 systems feature clays with the TOT structure, i.e., exposing the same tetrahedral type of surface to the solvent, and were thus associated with comparable ion distributions. In these cases, the upper and lower ion densities were summed. In the case of KGa-1, the TO clay, these surfaces were presented independently, as KGa-1-T for the tetrahedral siloxane surface and KGa-1-O for the octahedral hydroxyl surface. Each obtained density distribution was finally independently normalized.

The locations of peaks in the number densities are associated with characteristic ion adsorption modes. By separately plotting the number densities of Ba^2+^ and Na^+^, we identified common minima in distributions, that we took as criteria to define regions associated with each adsorption mode (see Figure S3, SI). By integrating each region, we could quantify the percentage of ions adsorbed in each mode. For this quantification, for SWy-simp., SWy-1, and IMt-1 systems, upper and lower number densities were averaged. For KGa-1, we only considered the distribution of ions on the tetrahedral side.

### 3.4 Visualization

Plots were produced with in-house software using the matplotlib^56^ Python package. Renderings of clay UC structures are made with VESTA.^57^ Simulation renderings were produced with VMD^44^ with the colors as follows, unless stated otherwise. Clay layers are shown with balls and sticks representations, atom colors are Al—pink, Mg—cyan, both Fe^II^ and Fe^II^—green, O—red, H—white. Ionic species are shown as van der Waals spheres of different colors: Ba^2+^—orange, Na^+^—blue, Cl^–^—green, Mg^2+^—gray, Ca^2+^—silver, K^+^—black. Bulk water molecules are not shown for clarity, individual molecules of water are shown in balls and sticks representation with O—red and H—white.

## 4 Results and Discussion

### 4.1 Construction of Accurate Clay Models

**Figure 4:**
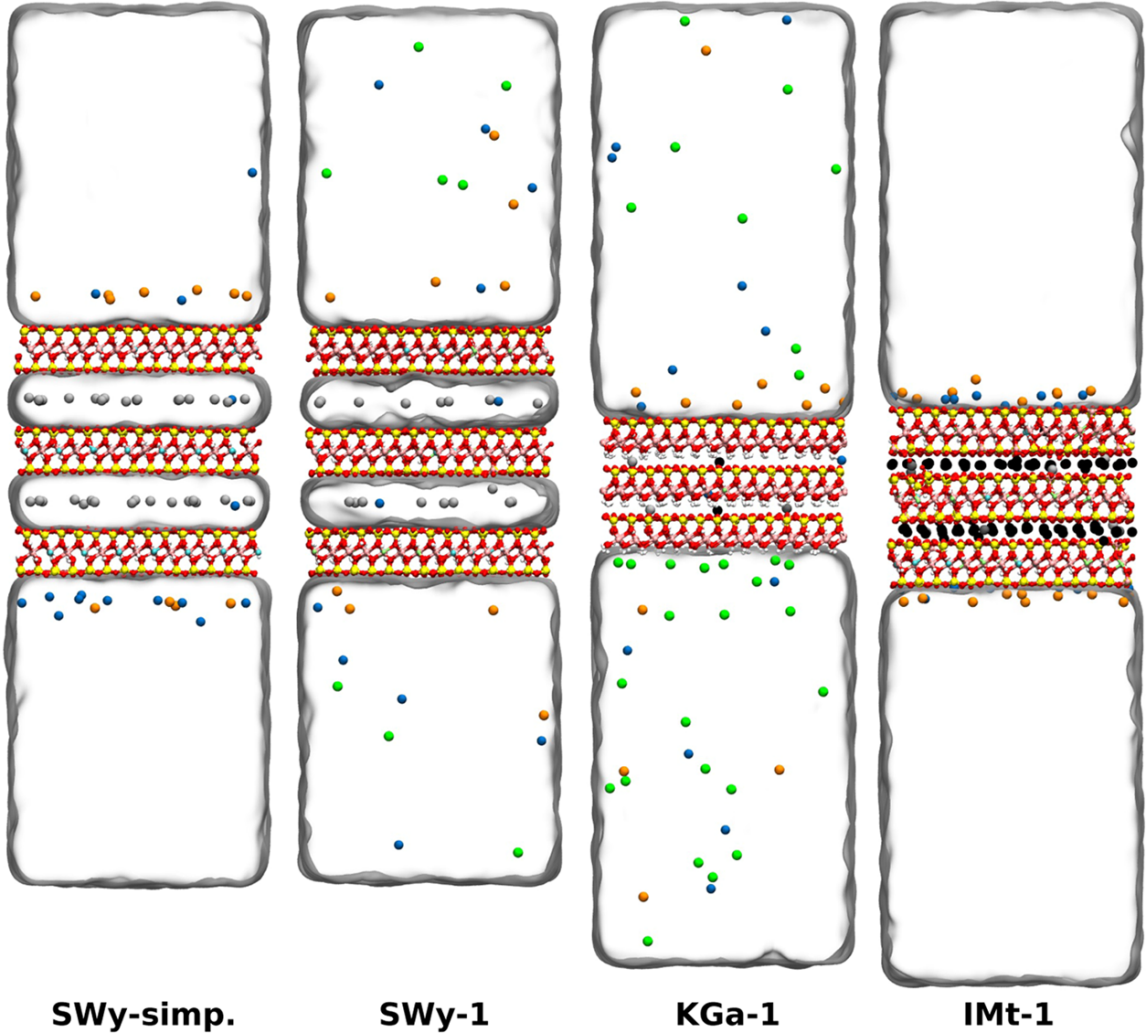
Side view of the four systems produced with *ClayCode* after equilibration. Clay layers in the middle, water is represented as transparent surfaces and ionic species are shown as spheres of different colors.

We used *ClayCode* to construct clay models with compositions closely matching experimental stoichiometries (see Table 4). The resulting clay systems produced by *ClayCode* are shown in Figure 4. The obtained SWy-1 model recapitulates the experimentally determined Wyoming montmorillonite (SWy-1/SWy-2/SWy-3) composition substantially better than previously used simplified models (SWy-simp.).^18,19^ Both the KGa-1 and IMt-1 models feature a slightly higher negative charge than their reference structures. We note that these reference structures feature a small inclusion of Ti^4+^ and trace amounts of Mn^2+^. Since no ClayFF force field parameters are currently available for these elements, these are not included in the *ClayCode*-produced structures. For Ti^4+^, we note that the experimental assignment in the reference structures may not always correspond to an isomorphic substitution. Both Dolcater et al. and Shoval et al. have observed strong bands of accessory anatase, TiO_2_, in Raman spectra of kaolinites.^58,59^ Specifically, Dolcater et al. found that 86% of Ti^4+^ in kaolinites is incorporated in the form of anatase, with Shoval et al. also noting minor incorporation of Ti^4+^ in the form of octahedral substitutions which are not expected to exceed 0.02 atoms UC^-1^.^59^*ClayCode* handling of these minor substitutions resulted in slightly elevated charges for IMt-1 and KGa-1 models with respect to their experimental counterparts.

### 4.2 Cation Adsorption on Two Wyoming Montmorillonite Models

To investigate the importance of using accurate clay models in simulations, we compared Na^+^ and Ba^2+^ adsorption onto the simplified Wyoming montmorillonite model (SWy-simp.), against the more realistic model (SWy-1) set up with *ClayCode* (see Table 4).

**Figure 5:**
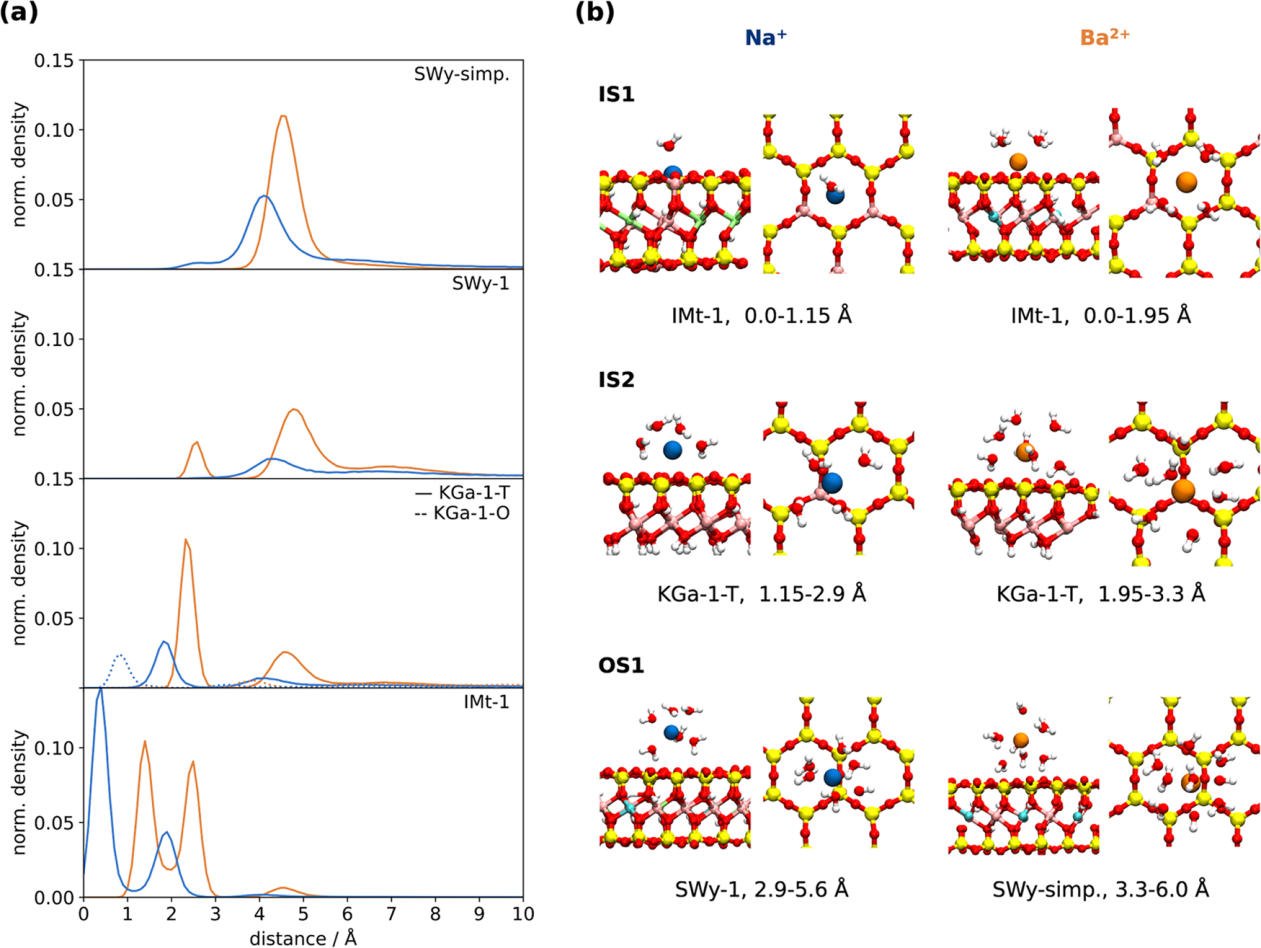
(a) Normalized linear densities of Na^+^ (blue) and Ba^2+^ (orange) ions for all simulated systems. The *x*-axis represents the distance of ions from the closest clay distribution peak (see Figure S2, SI). (b) For both Na^+^ and Ba^2+^ ions, representative snapshots of inner-shell (IS1, IS2) and outer-shell (OS1) adsorption modes. Water molecules in the first hydration shell of each ion are shown. Snapshots representing each peak in every simulation are presented in Figure S4, SI.

Comparing the two models, we find that the SWy-simp. features substantially higher adsorption for both Na^+^ and Ba^2+^ (see Figure 5a and Table 5). Specifically, SWy-simp. adsorbs 80% of total Na^+^, while SWy-1 adsorbs significantly less–only 36%. Similarly, for Ba^2+^, SWy-simp. adsorbs nearly all cations present (99.5%), while SWy-1 adsorbs a slightly smaller amount (82%). This difference is consistent with the difference in total charge between these two models (−1.0 |*e*| UC^–1^ for SWy-simp. and = −0.54 |*e*| UC^–1^ for SWy-1).

**Table 5:** Ionic Adsorption Capacities on Siloxane Surface Only, Split into the Adsorption Type Determined by the Distance Away from the Surface, and Selectivity for Ba^2+^ over Na^+^ for Each Clay System Modelled^a^

	ion adsorption per adsorption type (%)										
	Na^+^					Ba^2+^					
adsorption (distance, Å)	IS1 (0.0–1.15)	IS2 (1.15–2.9)	OS1 (2.9–5.6)	OS2 (5.6–10)	not ads.	IS1 (0.0–1.95)	IS2 (1.95–3.3)	OS1 (3.3–6.0)	OS2 (6.0–10)	not ads.	selectivity Ba^2+^/Na^+^
SWy-simp.	–	3.5	59.8	16.4	20.3	–	0.1	94.0	5.4	0.5	1.25
SWy-1	–	0.3	18.9	16.7	64.1	–	10.1	50.1	21.7	18.1	2.2
KGa-1-T	0.1	18.0	9.2	7.4	65.2	0.2	42.7	25.2	10.0	21.9	2.2
IMt-1	65.2	26.7	2.2	1.1	4.8	49.2	43.3	5.3	0.7	1.5	1.1

a For representative structures see Figure S4, SI.

These differences also mean that the selectivity for Ba^2+^ over Na^+^ is very different for these two clay models. SWy-simp. shows only a slight preference for Ba^2+^ over Na^+^ (1.25:1), while the realistic SWy-1 model is much more selective (2.2:1). The interactions of these cations with SWy-simp. were previously studied with metadynamics by Underwood et al. The study highlighted that unbiased MD was able to reproduce well the adsorption profiles for these two cations, and calculated their exchange equilibrium constant of 1.33, in agreement with the selectivity ratio predicted in this work for the same simplified montmorillonite model.^19^

There exist many studies determining experimental exchange equilibrium constants for montmorillonites/bentonites, owing to their applications as barriers for various heavy metal wastes. Older works report exchange constants between 1.1 and 2.3 for montmorillonites, though notably for slightly different varieties to the one in this work.^60,61^ A more recent study by Klinkenberg et al. has found that the exchange coefficients of Ba^2+^ for Na^+^ on Wyoming montmorillonite (SWy-1) to be concentration-dependent, increasing with increased ionic strength (2.46 for 0.3 M solution, 2.0 for under 0.02 M).^23^ This, again, is in perfect agreement with the findings from our simulation of the realistic SWy-1 clay.

The higher preference for divalent cation by the realistic SWy-1 can be attributed to the formation of the inner-hydration sphere (IS) complex in addition to the outer-hydration sphere (OS) complex, which is also observed for in the SWy-simp. model (peaks at 2.5 and 4.5 Å distance away from the surface, respectively, see Figure 5). The IS complex of Ba^2+^ is facilitated by the small number of Al substitutions in the T sheet of SWy-1. Interestingly, Zhang et al. observed via EXAFS that at high pH (i.e., not on the edge-site) and high concentrations, Ba^2+^ forms both IS and OS complexes on SWy-1 clay.^24^

Our comparison of cation adsorption on two models of Wyoming montmorillonite–one simplified used in previous works and one realistic assembled with *ClayCode*–highlights the importance of accounting for the finest structure features in clay minerals to truly gain atomic-level insight into experimental observables.

### 4.3 Interaction of Cations with Realistic Montmorillonite, Illite, and Kaolinite Models

In addition to Wyoming montmorillonite, we also investigated the effect of clay composition and structure for Na^+^ and Ba^2+^ adsorption on kaolinite KGa-1 and illite IMt-1 clays. Alike montmorilonite, illite is a 2:1 clay exposing two tetrahedral surfaces to the solvent. On the other hand, kaolinite is a 1:1 clay exposing one tetrahedral and one octahedral surface to the solvent. We first examine ion adsorption on the two KGa-1 surfaces. We note that experimentally it is near-impossible to distinguish what kaolinite surface participates in the adsorption, or to quantify the ratio of the exposed surfaces in the solution. However, with simulations we can easily separate these two surfaces. Figure 5 compares the adsorption of cations on the octahedral (KGa-1-O, dashed line) and on the tetrahedral (KGa-1-T, solid line) surfaces, showing that only minimal Na^+^ and no Ba^2+^ are adsorbed onto the KGa-1-O. For this reason, we omit the contribution of the octahedral surface from the adsorption capacity calculations presented in Table 5.

The behavior of the KGa-1 is similar to the realistic SWy-1 model, with the same adsorption preference for Ba^2+^ over Na^+^ of 2.2. Similarly, the adsorption of both cations is found above the tetrahedral Al substitution (see IS2 renderings on Figure 5b), which is slightly higher in KGa-1 than in SWy-1 (KGa-1 features 0.17 Al per 4 T positions, SWy-1 only 0.03 Al per 8, see Table 4).

For IMt-1, nearly all of the Na^+^ and Ba^2+^ ions were adsorbed, always forming IS complexes. In fact, we identified two IS complexes: IS2 at approximately 2.0 Å for Na^+^ and 2.5 Å for Ba^2+^ distance from the surface and IS1 much closer to the surface at just 0.5 Å for Na^+^ and 1.5 Å for Ba^2+^ (Figure 5). While IS2 is identical to the IS adsorption mode found on KGa-1 and SWy-1, where the cation sits directly above the tetrahedral Al substitution, IS1 is facilitated by the frequent proximate Al substitutions that act like pliers, holding the cation tightly between them (see IS1 on Figure 5). In the case of the smaller Na^+^ (ionic radius of 1.16 Å compared to 1.49 Å for Ba^2+^), ions nearly fully submerge into the silicate crown of the tetrahedral surface, with only one water coordinated above them. Ba^2+^ protrudes further, allowing four waters to sit above it. While the near-complete adsorption of cations on IMt-1 does not enable an accurate estimation of selectivity, in line with expectations, we observe a slightly higher preference for Ba^2+^.

Overall, our simulations, in agreement with experimental works, have shown that all modeled clays have a slight preference for Ba^2+^, resulting in over 70% of its removal from solution.^22,23^ Furthermore, leveraging accurate molecular models allowed us to gain atomistic insights into the specific adsorption mechanisms for each clay, highlighting how isomorphic substitutions determine their specific chemophysical properties.

## 5 Conclusions

In this work, we introduce *ClayCode* – a comprehensive and user-friendly software designed to facilitate and advance molecular modeling of clay materials. By enabling the construction of clay models that closely resemble their experimentally determined structures, *ClayCode* addresses the critical need for realistic and representative simulations that are vital for interpreting and predicting the behavior of clay minerals in varied scientific and engineering contexts.

Through the simulation of Na^+^ and Ba^2+^ ions adsorption on common clay minerals–Wyoming montmorillonite (both simplified and realistic models), Georgia kaolinite and Montana illite–we demonstrated that the structural accuracy of the models has a significant effect on simulation outcomes. For instance, our findings illustrate that a realistic representation of montmorillonite predicts cation adsorption with greater fidelity to experimental results, thereby revealing essential details about ion exchange dynamics that were not captured by simpler, idealized models. Another outcome enabled by setting up clay models with *ClayCode*, was the variability in ion adsorption efficiency and selectivity among different types of clays, all driven by subtle differences in their compositional and structural properties. This further reinforces the importance of employing accurate clay models for studies where molecular-level interactions govern the macroscopic properties.

Looking forward, while *ClayCode* has already proven its utility in modeling planar, hydrated clay systems, its flexible and modular design lays a robust foundation for future enhancements. The goals of forthcoming developments are to extend its capabilities to include pH-dependent edge sites and nonplanar geometries. Additionally, further development of an integrated analysis module within *ClayCode* will streamline the workflow from model construction to analysis, facilitating rapid and in-depth interpretation of simulation results.

In conclusion, *ClayCode* represents a significant step forward in computational clay mineral modeling, enabling researchers to build more accurate models that can better mimic real-world materials. This, in turn, enhances the reliability of simulations and the insights they can provide, supporting more informed decisions in the fields of environmental and material sciences.

## Data Availability

*ClayCode* is available open-source at github.com/Erastova-group/ClayCode, DOI: 10.5281/zenodo.11219451. A workshop material dedicated to ClayCode is also available: github.com/Erastova-group/ClayCode-workshop. Manual and Tutorials are available at claycode.readthedocs.io.

## Supporting Information Available

Number of research articles published per year mentioning “clay molecular dynamics”; raw normalized densities for Na^+^, Ba^2+^, and clay atoms; number density of Na^+^ and Ba^2+^ ions exposed to different surface; renderings of representative mechanisms of adsorption for Na^+^ and Ba^2+^ cations on the clays; simulation box dimensions after equilibration, number and type of inserted bulk ions, and interlayer and bulk solvent molecules for the simplified (SWy-simp.) and realistic SWy-1, IMt-1, and KGa-1 models (PDF)
ClayCode YAML and CSV input files used in this work (ZIP)
