# Supplementary material for: Modeling Realistic Clay Systems with ClayCode
Source: J Chem Theory Comput. 2024 Oct 15;20(21):9606–17. doi: 10.1021/acs.jctc.4c00987 (PMC11562070; doi:10.1021/acs.jctc.4c00987)
Supplement: Supplementary file 1 — ct4c00987_si_001.pdf [file ct4c00987_si_001.pdf]

Supporting Information: Modelling realistic clay systems with ClayCode

Hannah Pollak,† Matteo T. Degiacomi,‡ and Valentina Erastova*,†,¶

†*School of Chemistry, University of Edinburgh, Joseph Black Building, David Brewster Road, Edinburgh, EH9 3FJ, United Kingdom*

‡*Department of Physics, Durham University, South Road, Durham, DH1 3LE, United Kingdom*

¶*UK Centre for Astrobiology, School of Physics and Astronomy, University of Edinburgh, James Clerk Maxwell Building, Peter Guthrie Tait Road, Edinburgh, EH9 3FD, United Kingdom*

E-mail: valentina.erastova@ed.ac.uk

Supporting Information Available

Input files

YAML files - user inputs build specification for each clay type:

- `SWy-simplified.yaml`
- `SWy-1.yaml`
- `KGa-1.yaml`
- `IMt-1.yaml`

CSV file - Experimentally determined structure file: `exp_clay.csv`

These files are also available for download from `/paper/` directory within *ClayCode* at github.com/Erastova-group/ClayCode

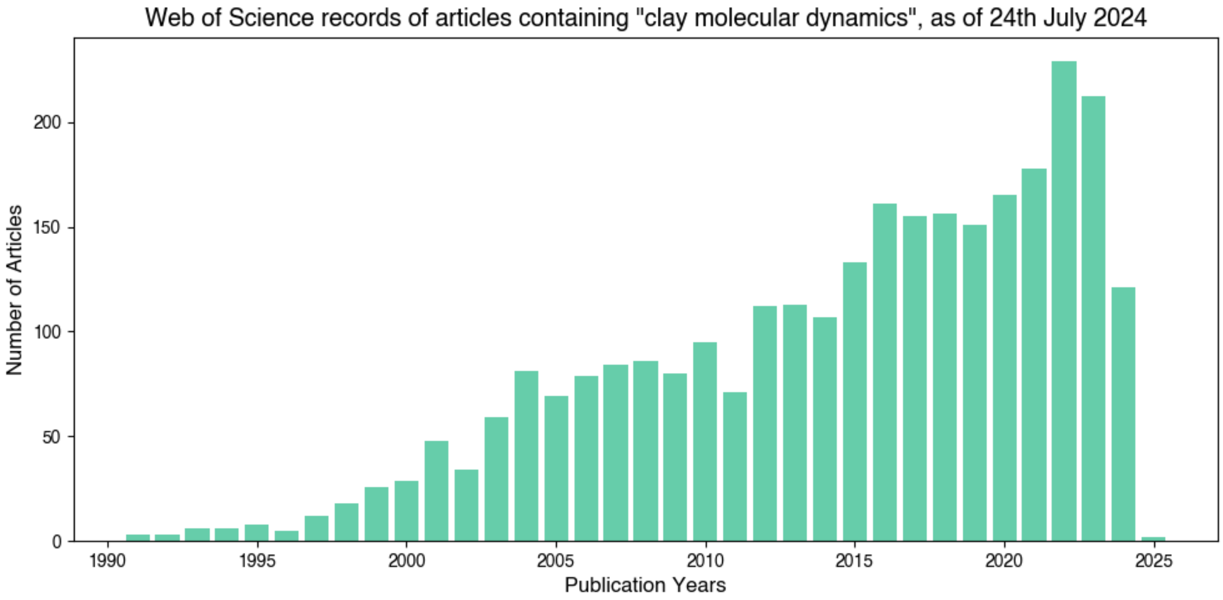

Figure S1: Number of research articles published per year mentioning "clay molecular dynamics", the record data is obtained from the Web of Science on the 24th July 2024.

Table S1: Simulation box dimensions after equilibration, number and type of inserted bulk ions, and interlayer (IL) and bulk solvent molecules for the simplified (SWy-simp.) and realistic SWy-1, IMt-1, and KGa-1 models.

Clay	Sim. box size (nm)			Bulk ions			Water molecules	
	x	y	z	Na^+	Ba^{2+}	Cl^-	IL	bulk
SWy-simp.	3.625	4.50	12.91	13	11	0	690	3557
SWy-1	3.63	4.51	12.80	9	9	8	590	3369
IMt-1	3.63	4.51	14.37	20	19	0	–	4270
KGa-1	3.62	4.49	14.10	12	12	30	–	4228

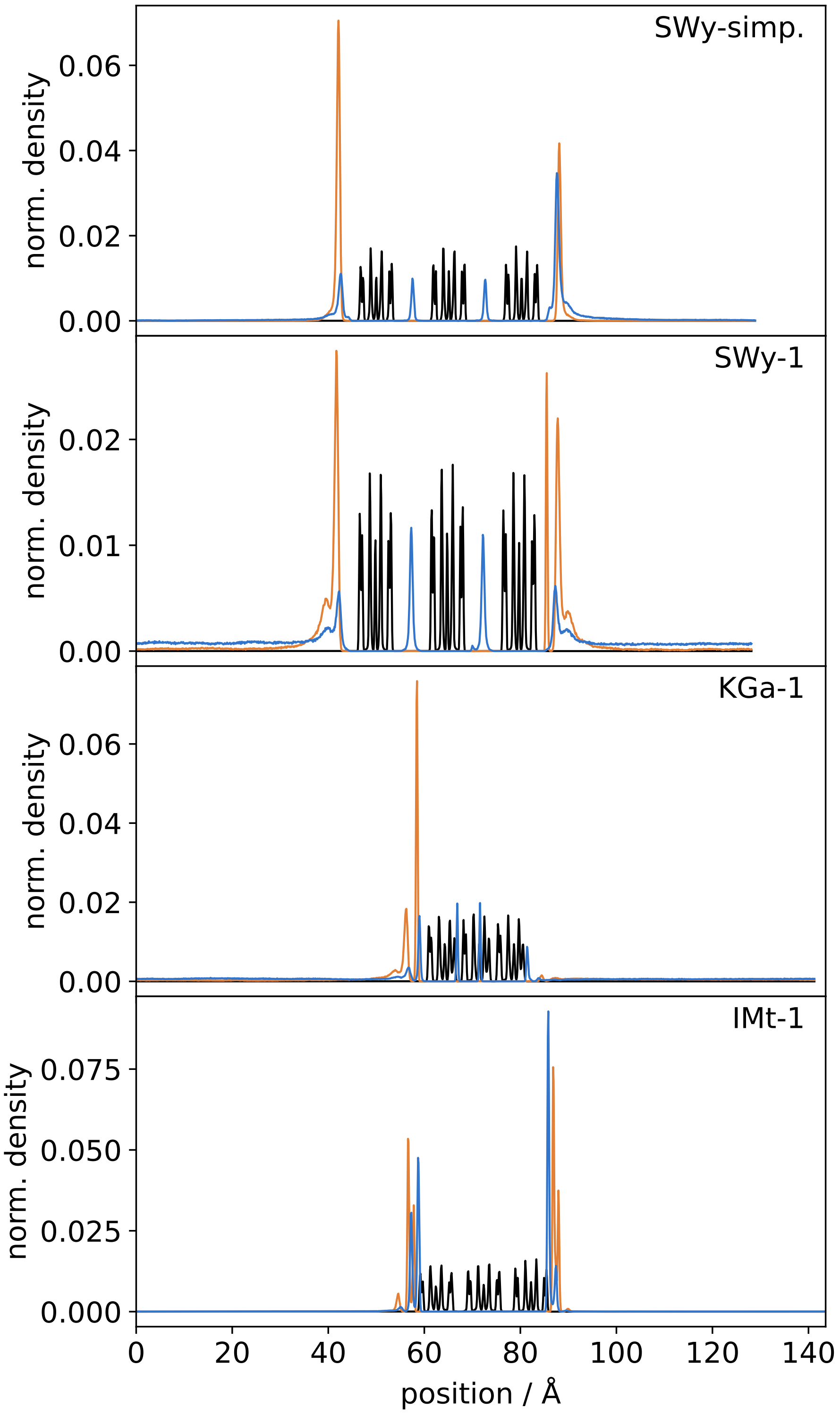

Figure S2: Raw normalized densities for Na^{+} (blue) and Ba^{2+} (orange) and clay (black) atoms. The x-axis represents absolute position in the z-axis of each simulation box.

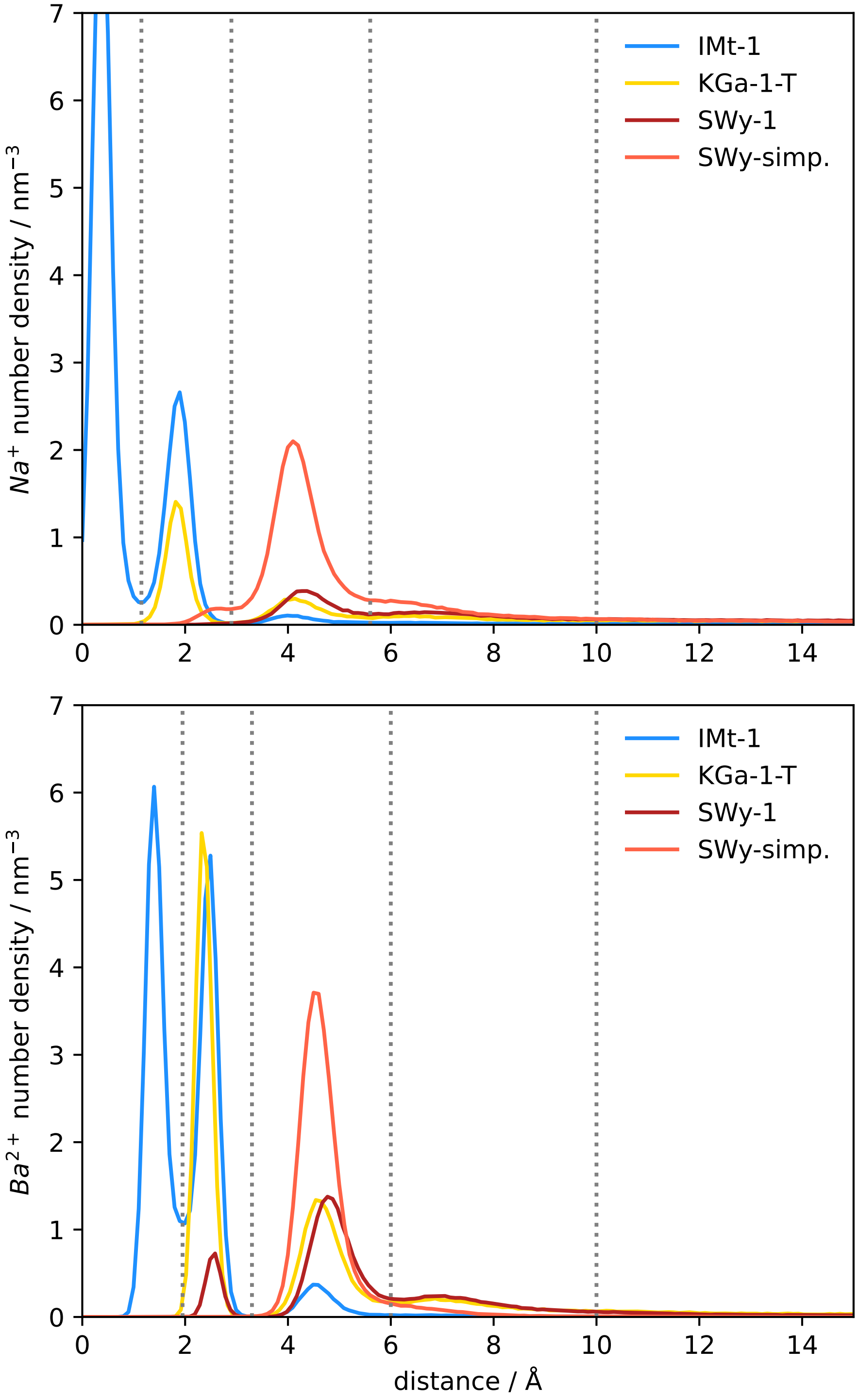

Figure S3: Number density of Na^+ (above) and Ba^{2+} (below) ions exposed to different surfaces. Gray dashed lines represent the cutoff distances adopted to separate the partial densities into regions associated with different adsorption modes (as listed in Table 3 of the main text). For Na^+: IS1 is at 0.00 - 1.15 Å away from the surface, IS2 at 1.15 - 2.9 Å, OS1 2.9 - 5.6 Å, OS2 5.6 - 10.0 Å. For Ba^{2+}: IS1 is at 0.0 - 1.95 Å, IS2 at 1.95 - 3.3 Å, OS1 at 3.3 - 6.0 Å, OS2 at 6.0 - 10.0 Å. Beyond 10 Å distance away from the surface cations are in the bulk, i.e., not adsorbed.

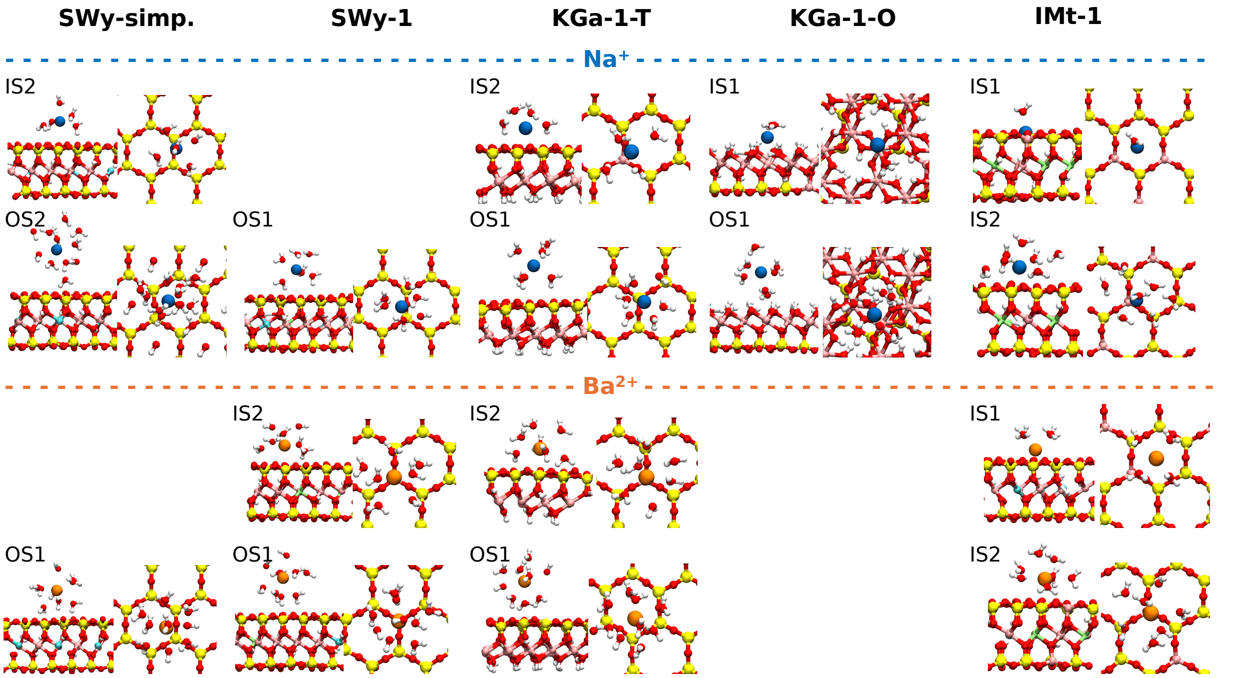

Figure S4: Renderings of representative mechanisms of adsorption for Na^+ and Ba^{2+} cations on the clays. Only the water molecules forming hydration shell around adsorbed ions are shown. The adsorption mechanisms are defined by the linear density profiles (Fig. S3) and are as follows, for Na^+: IS1 is at 0.00 - 1.15 Å away from the surface, IS2 at 1.15 - 2.9 Å, OS1 2.9 - 5.6 Å, OS2 5.6 - 10.0 Å; for Ba^{2+}: IS1 is at 0.0 - 1.95 Å, IS2 at 1.95 - 3.3 Å, OS1 at 3.3 - 6.0 Å, OS2 at 6.0 - 10.0 Å.
